# (7*E*)-5-Benzyl-7-(2-chloro­benzyl­idene)-3-(2-chloro­phen­yl)-2-phenyl-3,3a,4,5,6,7-hexa­hydro-2*H*-pyrazolo­[4,3-*c*]pyridine

**DOI:** 10.1107/S1600536810023317

**Published:** 2010-06-23

**Authors:** N. S. Karthikeyan, B. Uma Mahesh, K. Sathiyanarayanan, P. Raghavaiah, R. S. Rathore

**Affiliations:** a Chemistry Division, School of Science and Humanities, VIT University, Vellore 632 014, India; b School of Chemistry, University of Hyderabad, Hyderabad 500 046, India; c Bioinformatics Infrastructure Facility, Department of Biotechnology, School of Life Science, University of Hyderabad, Hyderabad 500 046, India

## Abstract

In the title 2*H*-pyrazolo­[4,3-*c*]pyridine derivative, C_32_H_27_Cl_2_N_3_, the dihydro­pyrazole ring adopts an envelope conformation and the piperidine fused ring a twisted-chair conformation. Two short intra­molecular C—H⋯Cl contacts are observed. The crystal packing is characterized by dimeric C—Cl⋯π inter­actions  involving the 5-benzyl ring, with Cl⋯centroid and closest atomic Cl⋯π distances of 3.778 (2) and 3.366 (4) Å, respectively.

## Related literature

For the anti-inflammatory activity of 2*H*-pyrazolo­[4,3-*c*]pyridine derivatives, see Krapcho & Turk (1975 ▶). For π-halogen-dimer inter­actions and their role in host–guest chemistry, see: Noman *et al.* (2004 ▶); Nagaraj *et al.* (2005 ▶). 
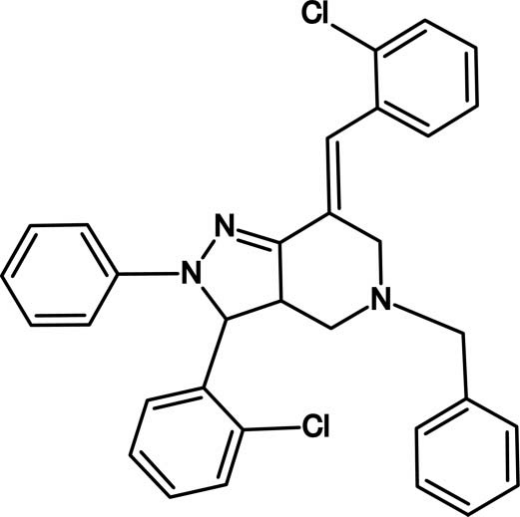


## Experimental

### 

#### Crystal data

C_32_H_27_Cl_2_N_3_
*M* *_r_* = 524.47
Monoclinic, 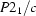

*a* = 13.7117 (7) Å
*b* = 15.4451 (6) Å
*c* = 13.6896 (9) Å
β = 113.135 (7)°
*V* = 2666.0 (2) Å^3^
*Z* = 4
Mo *K*α radiation
μ = 0.27 mm^−1^
*T* = 294 K
0.36 × 0.26 × 0.22 mm

#### Data collection

Oxford Diffraction Xcalibur Eos Gemini diffractometer
Absorption correction: multi-scan (*CrysAlis PRO*; Oxford Diffraction, 2009 ▶) *T*_min_ = 0.909, *T*_max_ = 0.943
11774 measured reflections
5436 independent reflections
2483 reflections with *I* > 2σ(*I*)
*R*_int_ = 0.049

#### Refinement

*R*[*F* ^2^ > 2σ(*F* ^2^)] = 0.049
*wR*(*F* ^2^) = 0.098
*S* = 0.83
5436 reflections
334 parameters
H-atom parameters constrained
Δρ_max_ = 0.17 e Å^−3^
Δρ_min_ = −0.23 e Å^−3^

### 

Data collection: *CrysAlis PRO* (Oxford Diffraction, 2009 ▶); cell refinement: *CrysAlis PRO*; data reduction: *CrysAlis PRO*; program(s) used to solve structure: *SHELXS97* (Sheldrick, 2008 ▶); program(s) used to refine structure: *SHELXL97* (Sheldrick, 2008 ▶); molecular graphics: *ORTEP-3* (Farrugia, 1997 ▶) and *PLATON* (Spek, 2009 ▶); software used to prepare material for publication: *SHELXL97* and *PLATON*.

## Supplementary Material

Crystal structure: contains datablocks global, I. DOI: 10.1107/S1600536810023317/ng2791sup1.cif

Structure factors: contains datablocks I. DOI: 10.1107/S1600536810023317/ng2791Isup2.hkl

Additional supplementary materials:  crystallographic information; 3D view; checkCIF report

## Figures and Tables

**Table 1:** Hydrogen-bond geometry (Å, °)

*D*—H⋯*A*	*D*—H	H⋯*A*	*D*⋯*A*	*D*—H⋯*A*
C3—H3⋯Cl1	0.98	2.61	3.101 (2)	111
C27—H27⋯Cl2	0.93	2.68	3.043 (3)	104

**Fig. 1.**
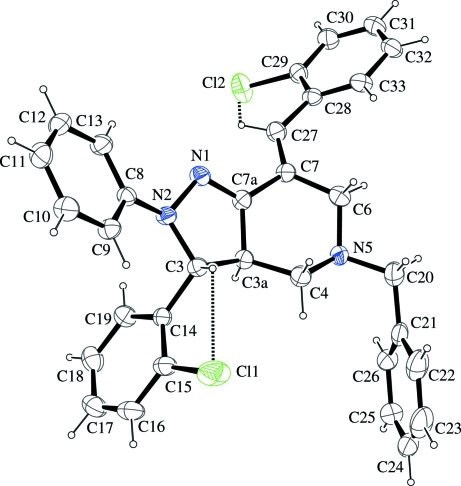
A view of (I) with non-H atoms shown as probability ellipsoids at 30% levels (Farrugia, 1997). The radii of H atoms are on an arbitrary scale. Dashed lines indicate short intra-molecular C—H···Cl contacts.

**Fig. 2.**
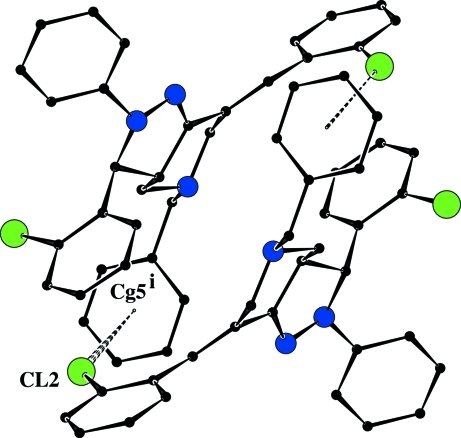
Dimeric subunits linked by C—Halogen···π interaction in (I). Cg5 is the centroid of (C21—C26) ring.

## supplementary crystallographic information

### Comment

Derivatives of 2*H*-pyrazolo[4,3-*c*]pyridine have been tested for anti-inflammatory activity (Krapcho & Turk, 1975). A search in Cambridge Structural Database (version 5.31) for such compounds retrieved zero hits. With a purpose to study hitherto unexplored structures of these compounds, we here report the synthesis and structural investigations on, 5-benzyl-(7E)-7-(2-chlorobenzylidene)-3-(2-chlorophenyl)-2-phenyl- 3,3a,4,5,6,7-hexahydro-2H-pyrazolo[4,3-c]pyridine, (I).

The structure of (I) with adopted atomic numbering scheme is shown in Fig 1. (I) is a racemic mixture. In the reported model, the stereogenic centers C3 and C3A possess *R*-configurations. The five-membered dihydropyrazole ring (N1/N2/C3/C3A/C7A) adopt an envelope conformation with atom C3 at the flap of the envelope (Ring puckering parameters are: q2 = 0.204 (2) Å, φ2 = 248.3 (6)°). The adjacent 6-membered piperidine ring (C3A/C4/N5/C6/C7/C7A) assumes a chair conformation which is substantially twisted from ideal geometry. The puckering parameters are as follows: q2 = 0.189 (2) Å, q3 = -0.468 (2) Å, θ = 158.0 (2)°, φ = 209.5 (8)°, and total puckering amplitude, Q = 0.505 (2) Å.

Two short intra-molecular contacts C3—H3···Cl1 and C27—H27···Cl2 were observed (Table 1). Intermolecular C—Halogen···π contact stabilizes the dimeric units in (I) (Fig 2). A dimer is formed by C29—Cl2···*Cg*5^i^ [symmetry code (i): 1 - *x*, 1 - *y*, 1 - *z*]. The Cl2..*Cg*5 distance and C29—Cl2···*Cg*5 angle are 3.778 (2)Å and 141.2 (1)° respectively, whereas the minimum atomic distance in Cl2···π is 3.366 (4) Å. *Cg*5 is the centroid of (C21–C26) ring. The C—Halogen···π dimeric interactions [also referred as PHD; π-halogen-dimer interactions (Noman *et al.* 2004)] have been shown recently, to play an important role in host–guest chemistry (Nagaraj *et al.*, 2005; references therein).

### Experimental

1-benzyl-3, 5-dibenzylidenepiperidin-4-one (0.003 mol) and phenyl hydrazine (0.003 mol) were dissolved in 2-propanol. The reaction mixture was refluxed for 1–2 h on a water bath and tested with TLC at regular intervals for completeness of reaction. Following that, the resulting mixture was cooled and poured into crushed ice. The solid so obtained was separated, washed with water and subjected to column chromatography using ethyl acetate and n-hexane. Final yield 89%, m.p. 153–155° C. Suitable single crystals for data collection were grown from ethanol and tetrahydrofuran mixture in 1:1 ratio.

### Refinement

H atoms were placed in their stereochemically expected positions and refined with the riding options. The distances with hydrogen atoms are: C(aromatic/*sp*^2^)—H = 0.93 Å, *C*(methylene)—H = 0.97 Å, C(methine)—H = 0.98 Å, and *U*_iso_ = 1.2 *U*_eq_(parent atom).

### Crystal data

**Table:**

C_32_H_27_Cl_2_N_3_	*F*(000) = 1096
*M**_r_* = 524.47	*D*_x_ = 1.307 Mg m^−^^3^
Monoclinic, *P*2_1_/*c*	Melting point: 427(2) K
Hall symbol: -P 2ybc	Mo *K*α radiation, λ = 0.71073 Å
*a* = 13.7117 (7) Å	Cell parameters from 2998 reflections
*b* = 15.4451 (6) Å	θ = 2.6–29.1°
*c* = 13.6896 (9) Å	µ = 0.27 mm^−^^1^
β = 113.135 (7)°	*T* = 294 K
*V* = 2666.0 (2) Å^3^	Plate, colorless
*Z* = 4	0.36 × 0.26 × 0.22 mm

### Data collection

**Table:**

Oxford Diffraction Xcalibur Eos Gemini diffractometer	5436 independent reflections
Radiation source: Enhance (Mo) X-ray Source	2483 reflections with *I* > 2σ(*I*)
graphite	*R*_int_ = 0.049
Detector resolution: 16.3291 pixels mm^-1^	θ_max_ = 26.4°, θ_min_ = 2.6°
ω scan	*h* = −14→17
Absorption correction: multi-scan (*CrysAlis PRO*; Oxford Diffraction, 2009)	*k* = −19→17
*T*_min_ = 0.909, *T*_max_ = 0.943	*l* = −15→17
11774 measured reflections	

### Refinement

**Table:**

Refinement on *F*^2^	Primary atom site location: structure-invariant direct methods
Least-squares matrix: full	Secondary atom site location: difference Fourier map
*R*[*F*^2^ > 2σ(*F*^2^)] = 0.049	Hydrogen site location: inferred from neighbouring sites
*wR*(*F*^2^) = 0.098	H-atom parameters constrained
*S* = 0.83	*w* = 1/[σ^2^(*F*_o_^2^) + (0.0389*P*)^2^] where *P* = (*F*_o_^2^ + 2*F*_c_^2^)/3
5436 reflections	(Δ/σ)_max_ < 0.001
334 parameters	Δρ_max_ = 0.17 e Å^−^^3^
0 restraints	Δρ_min_ = −0.23 e Å^−^^3^

### Special details

**Table:**

Experimental. *CrysAlis PRO*, Oxford Diffraction Ltd., Version 1.171.33.55 (release 05–01-2010 CrysAlis171. NET) Empirical absorption correction using spherical harmonics, implemented in SCALE3 ABSPACK scaling algorithm.
Geometry. All e.s.d.'s (except the e.s.d. in the dihedral angle between two l.s. planes) are estimated using the full covariance matrix. The cell e.s.d.'s are taken into account individually in the estimation of e.s.d.'s in distances, angles and torsion angles; correlations between e.s.d.'s in cell parameters are only used when they are defined by crystal symmetry. An approximate (isotropic) treatment of cell e.s.d.'s is used for estimating e.s.d.'s involving l.s. planes.
Refinement. Refinement of *F*^2^ against ALL reflections. The weighted *R*-factor *wR* and goodness of fit *S* are based on *F*^2^, conventional *R*-factors *R* are based on *F*, with *F* set to zero for negative *F*^2^. The threshold expression of *F*^2^ > σ(*F*^2^) is used only for calculating *R*-factors(gt) *etc*. and is not relevant to the choice of reflections for refinement. *R*-factors based on *F*^2^ are statistically about twice as large as those based on *F*, and *R*- factors based on ALL data will be even larger.

### Fractional atomic coordinates and isotropic or equivalent isotropic displacement parameters (Å^2^)

**Table:**

	*x*	*y*	*z*	*U*_iso_*/*U*_eq_	
C3	0.68994 (17)	0.48100 (13)	0.39914 (17)	0.0384 (6)	
H3	0.7343	0.4316	0.3979	0.046*	
C3A	0.60177 (16)	0.44975 (13)	0.43353 (18)	0.0372 (6)	
H3A	0.5847	0.4958	0.4735	0.045*	
C4	0.62227 (17)	0.36610 (14)	0.49646 (19)	0.0466 (7)	
H4A	0.6489	0.3225	0.4623	0.056*	
H4B	0.6753	0.3756	0.5675	0.056*	
C6	0.44171 (18)	0.31461 (13)	0.39584 (19)	0.0483 (7)	
H6A	0.3770	0.2970	0.4031	0.058*	
H6B	0.4665	0.2659	0.3671	0.058*	
C7	0.41706 (18)	0.38915 (13)	0.31845 (19)	0.0399 (6)	
C7A	0.51168 (17)	0.43857 (14)	0.32849 (19)	0.0396 (6)	
C8	0.67598 (17)	0.52120 (13)	0.21565 (19)	0.0372 (6)	
C9	0.78552 (18)	0.51570 (14)	0.24683 (19)	0.0440 (6)	
H9	0.8286	0.5028	0.3170	0.053*	
C10	0.8300 (2)	0.52943 (15)	0.1736 (2)	0.0538 (7)	
H10	0.9033	0.5261	0.1954	0.065*	
C11	0.7685 (2)	0.54792 (15)	0.0691 (2)	0.0565 (7)	
H11	0.7992	0.5561	0.0202	0.068*	
C12	0.6598 (2)	0.55403 (15)	0.0385 (2)	0.0563 (7)	
H12	0.6173	0.5671	−0.0317	0.068*	
C13	0.61374 (19)	0.54113 (14)	0.1102 (2)	0.0491 (7)	
H13	0.5406	0.5458	0.0882	0.059*	
C14	0.75983 (17)	0.55254 (13)	0.46627 (18)	0.0371 (6)	
C15	0.86179 (18)	0.53885 (15)	0.54037 (19)	0.0496 (7)	
C16	0.9251 (2)	0.60585 (19)	0.5984 (2)	0.0626 (8)	
H16	0.9935	0.5946	0.6474	0.075*	
C17	0.8869 (2)	0.68811 (19)	0.5834 (2)	0.0629 (8)	
H17	0.9297	0.7336	0.6211	0.075*	
C18	0.7853 (2)	0.70400 (16)	0.5126 (2)	0.0603 (8)	
H18	0.7584	0.7601	0.5038	0.072*	
C19	0.72281 (19)	0.63714 (15)	0.4546 (2)	0.0494 (7)	
H19	0.6542	0.6489	0.4064	0.059*	
C20	0.5425 (2)	0.25732 (14)	0.5690 (2)	0.0565 (7)	
H20A	0.5710	0.2125	0.5381	0.068*	
H20B	0.4757	0.2366	0.5688	0.068*	
C21	0.6183 (2)	0.27240 (15)	0.6818 (2)	0.0503 (7)	
C22	0.7124 (2)	0.22752 (18)	0.7258 (3)	0.0785 (10)	
H22	0.7295	0.1863	0.6855	0.094*	
C23	0.7823 (3)	0.2434 (2)	0.8303 (4)	0.0997 (14)	
H23	0.8460	0.2132	0.8594	0.120*	
C24	0.7569 (3)	0.3035 (2)	0.8899 (3)	0.0999 (14)	
H24	0.8035	0.3141	0.9596	0.120*	
C25	0.6636 (3)	0.34798 (18)	0.8477 (2)	0.0777 (9)	
H25	0.6462	0.3886	0.8884	0.093*	
C26	0.5955 (2)	0.33212 (16)	0.7442 (2)	0.0601 (8)	
H26	0.5322	0.3628	0.7156	0.072*	
C27	0.32178 (17)	0.41049 (14)	0.2455 (2)	0.0456 (6)	
H27	0.3200	0.4618	0.2092	0.055*	
C28	0.21940 (17)	0.36564 (15)	0.21350 (18)	0.0413 (6)	
C29	0.12329 (18)	0.41044 (14)	0.1697 (2)	0.0454 (6)	
C30	0.02629 (18)	0.36958 (16)	0.1313 (2)	0.0552 (7)	
H30	−0.0359	0.4017	0.1019	0.066*	
C31	0.0221 (2)	0.28080 (17)	0.1368 (2)	0.0593 (8)	
H31	−0.0429	0.2524	0.1113	0.071*	
C32	0.1142 (2)	0.23457 (15)	0.1800 (2)	0.0570 (8)	
H32	0.1115	0.1746	0.1845	0.068*	
C33	0.21102 (19)	0.27578 (15)	0.21688 (19)	0.0507 (7)	
H33	0.2726	0.2428	0.2448	0.061*	
N1	0.52729 (14)	0.47054 (11)	0.24903 (16)	0.0431 (5)	
N2	0.62818 (14)	0.51001 (11)	0.28892 (15)	0.0403 (5)	
N5	0.52271 (14)	0.33591 (11)	0.50228 (15)	0.0428 (5)	
Cl1	0.91528 (6)	0.43491 (4)	0.56217 (7)	0.0870 (3)	
Cl2	0.12374 (5)	0.52302 (4)	0.16351 (7)	0.0764 (3)	

### Atomic displacement parameters (Å^2^)

**Table:**

	*U*^11^	*U*^22^	*U*^33^	*U*^12^	*U*^13^	*U*^23^
C3	0.0318 (12)	0.0456 (13)	0.0375 (14)	0.0018 (11)	0.0133 (12)	0.0007 (12)
C3A	0.0334 (13)	0.0410 (14)	0.0401 (14)	−0.0001 (10)	0.0174 (12)	−0.0006 (12)
C4	0.0388 (14)	0.0504 (15)	0.0494 (16)	−0.0005 (11)	0.0161 (14)	0.0036 (13)
C6	0.0433 (15)	0.0498 (15)	0.0512 (17)	−0.0036 (12)	0.0179 (14)	−0.0057 (13)
C7	0.0361 (14)	0.0483 (14)	0.0384 (15)	−0.0021 (11)	0.0179 (13)	−0.0016 (12)
C7A	0.0332 (14)	0.0460 (14)	0.0429 (16)	0.0006 (11)	0.0187 (13)	0.0023 (12)
C8	0.0335 (14)	0.0410 (13)	0.0380 (14)	−0.0044 (11)	0.0152 (13)	−0.0030 (12)
C9	0.0394 (15)	0.0571 (15)	0.0380 (15)	0.0010 (12)	0.0180 (13)	−0.0014 (12)
C10	0.0424 (15)	0.0719 (17)	0.0555 (18)	−0.0033 (13)	0.0281 (16)	−0.0068 (15)
C11	0.0635 (19)	0.0644 (17)	0.0556 (19)	−0.0102 (14)	0.0384 (17)	−0.0052 (15)
C12	0.0599 (19)	0.0697 (17)	0.0403 (16)	−0.0072 (14)	0.0208 (16)	0.0048 (14)
C13	0.0379 (14)	0.0639 (17)	0.0440 (16)	−0.0046 (12)	0.0143 (14)	0.0015 (14)
C14	0.0351 (13)	0.0438 (14)	0.0352 (14)	−0.0031 (11)	0.0167 (12)	−0.0005 (12)
C15	0.0405 (14)	0.0607 (16)	0.0414 (15)	−0.0023 (13)	0.0095 (14)	−0.0021 (13)
C16	0.0460 (16)	0.084 (2)	0.0463 (18)	−0.0079 (16)	0.0063 (15)	−0.0097 (16)
C17	0.063 (2)	0.074 (2)	0.0553 (19)	−0.0245 (16)	0.0269 (17)	−0.0207 (16)
C18	0.070 (2)	0.0479 (15)	0.072 (2)	−0.0065 (14)	0.0376 (19)	−0.0086 (15)
C19	0.0418 (15)	0.0516 (16)	0.0548 (17)	0.0009 (13)	0.0187 (14)	−0.0034 (14)
C20	0.0609 (17)	0.0449 (15)	0.0643 (19)	−0.0002 (13)	0.0252 (17)	0.0074 (14)
C21	0.0457 (16)	0.0430 (15)	0.0597 (19)	0.0010 (13)	0.0179 (16)	0.0206 (15)
C22	0.063 (2)	0.0688 (19)	0.102 (3)	0.0148 (16)	0.031 (2)	0.037 (2)
C23	0.051 (2)	0.090 (3)	0.130 (4)	0.010 (2)	0.007 (3)	0.064 (3)
C24	0.073 (3)	0.095 (3)	0.090 (3)	−0.032 (2)	−0.013 (2)	0.049 (2)
C25	0.083 (2)	0.077 (2)	0.057 (2)	−0.0231 (18)	0.010 (2)	0.0085 (18)
C26	0.0543 (18)	0.0576 (17)	0.0574 (19)	−0.0043 (14)	0.0100 (17)	0.0125 (16)
C27	0.0397 (15)	0.0499 (14)	0.0486 (17)	−0.0030 (12)	0.0189 (14)	0.0001 (13)
C28	0.0369 (14)	0.0516 (15)	0.0361 (15)	−0.0030 (12)	0.0152 (13)	−0.0033 (12)
C29	0.0399 (15)	0.0487 (14)	0.0493 (16)	−0.0047 (12)	0.0193 (14)	0.0013 (13)
C30	0.0377 (15)	0.0598 (17)	0.0600 (19)	0.0001 (12)	0.0104 (15)	0.0037 (15)
C31	0.0448 (16)	0.0624 (18)	0.0586 (19)	−0.0144 (14)	0.0072 (16)	−0.0048 (15)
C32	0.0527 (17)	0.0477 (15)	0.0564 (18)	−0.0077 (13)	0.0060 (16)	−0.0058 (14)
C33	0.0448 (16)	0.0528 (16)	0.0465 (16)	−0.0005 (12)	0.0093 (14)	−0.0077 (13)
N1	0.0299 (11)	0.0542 (12)	0.0451 (13)	−0.0051 (9)	0.0145 (11)	−0.0001 (11)
N2	0.0287 (11)	0.0570 (12)	0.0350 (12)	−0.0049 (9)	0.0124 (10)	0.0001 (10)
N5	0.0400 (12)	0.0443 (11)	0.0434 (12)	−0.0051 (9)	0.0156 (11)	0.0044 (10)
Cl1	0.0653 (5)	0.0766 (5)	0.0839 (6)	0.0217 (4)	−0.0087 (5)	0.0038 (4)
Cl2	0.0549 (4)	0.0535 (4)	0.1194 (7)	0.0007 (3)	0.0327 (5)	0.0127 (4)

### Geometric parameters (Å, °)

**Table:**

C3—N2	1.480 (3)	C17—C18	1.372 (3)
C3—C14	1.514 (3)	C17—H17	0.9300
C3—C3A	1.537 (3)	C18—C19	1.377 (3)
C3—H3	0.9800	C18—H18	0.9300
C3A—C7A	1.493 (3)	C19—H19	0.9300
C3A—C4	1.517 (3)	C20—N5	1.478 (3)
C3A—H3A	0.9800	C20—C21	1.503 (3)
C4—N5	1.474 (3)	C20—H20A	0.9700
C4—H4A	0.9700	C20—H20B	0.9700
C4—H4B	0.9700	C21—C26	1.373 (3)
C6—N5	1.481 (3)	C21—C22	1.377 (3)
C6—C7	1.510 (3)	C22—C23	1.397 (4)
C6—H6A	0.9700	C22—H22	0.9300
C6—H6B	0.9700	C23—C24	1.368 (5)
C7—C27	1.337 (3)	C23—H23	0.9300
C7—C7A	1.464 (3)	C24—C25	1.364 (4)
C7A—N1	1.287 (3)	C24—H24	0.9300
C8—C13	1.392 (3)	C25—C26	1.379 (3)
C8—C9	1.393 (3)	C25—H25	0.9300
C8—N2	1.408 (3)	C26—H26	0.9300
C9—C10	1.379 (3)	C27—C28	1.470 (3)
C9—H9	0.9300	C27—H27	0.9300
C10—C11	1.375 (3)	C28—C33	1.395 (3)
C10—H10	0.9300	C28—C29	1.398 (3)
C11—C12	1.383 (3)	C29—C30	1.376 (3)
C11—H11	0.9300	C29—Cl2	1.741 (2)
C12—C13	1.375 (3)	C30—C31	1.376 (3)
C12—H12	0.9300	C30—H30	0.9300
C13—H13	0.9300	C31—C32	1.367 (3)
C14—C15	1.383 (3)	C31—H31	0.9300
C14—C19	1.388 (3)	C32—C33	1.376 (3)
C15—C16	1.383 (3)	C32—H32	0.9300
C15—Cl1	1.741 (2)	C33—H33	0.9300
C16—C17	1.359 (3)	N1—N2	1.411 (2)
C16—H16	0.9300		
			
N2—C3—C14	111.73 (17)	C18—C17—H17	120.1
N2—C3—C3A	101.74 (17)	C17—C18—C19	120.2 (2)
C14—C3—C3A	115.45 (19)	C17—C18—H18	119.9
N2—C3—H3	109.2	C19—C18—H18	119.9
C14—C3—H3	109.2	C18—C19—C14	121.5 (2)
C3A—C3—H3	109.2	C18—C19—H19	119.2
C7A—C3A—C4	110.32 (17)	C14—C19—H19	119.2
C7A—C3A—C3	101.19 (18)	N5—C20—C21	113.11 (18)
C4—C3A—C3	116.68 (18)	N5—C20—H20A	109.0
C7A—C3A—H3A	109.4	C21—C20—H20A	109.0
C4—C3A—H3A	109.4	N5—C20—H20B	109.0
C3—C3A—H3A	109.4	C21—C20—H20B	109.0
N5—C4—C3A	109.35 (17)	H20A—C20—H20B	107.8
N5—C4—H4A	109.8	C26—C21—C22	118.0 (3)
C3A—C4—H4A	109.8	C26—C21—C20	120.6 (2)
N5—C4—H4B	109.8	C22—C21—C20	121.5 (3)
C3A—C4—H4B	109.8	C21—C22—C23	120.5 (3)
H4A—C4—H4B	108.3	C21—C22—H22	119.8
N5—C6—C7	113.30 (17)	C23—C22—H22	119.8
N5—C6—H6A	108.9	C24—C23—C22	119.8 (3)
C7—C6—H6A	108.9	C24—C23—H23	120.1
N5—C6—H6B	108.9	C22—C23—H23	120.1
C7—C6—H6B	108.9	C25—C24—C23	120.3 (3)
H6A—C6—H6B	107.7	C25—C24—H24	119.8
C27—C7—C7A	120.7 (2)	C23—C24—H24	119.8
C27—C7—C6	126.6 (2)	C24—C25—C26	119.3 (3)
C7A—C7—C6	112.69 (19)	C24—C25—H25	120.4
N1—C7A—C7	123.8 (2)	C26—C25—H25	120.4
N1—C7A—C3A	114.9 (2)	C21—C26—C25	122.1 (3)
C7—C7A—C3A	121.2 (2)	C21—C26—H26	119.0
C13—C8—C9	118.6 (2)	C25—C26—H26	119.0
C13—C8—N2	119.9 (2)	C7—C27—C28	130.2 (2)
C9—C8—N2	121.5 (2)	C7—C27—H27	114.9
C10—C9—C8	120.0 (2)	C28—C27—H27	114.9
C10—C9—H9	120.0	C33—C28—C29	115.5 (2)
C8—C9—H9	120.0	C33—C28—C27	122.7 (2)
C11—C10—C9	121.4 (2)	C29—C28—C27	121.6 (2)
C11—C10—H10	119.3	C30—C29—C28	122.9 (2)
C9—C10—H10	119.3	C30—C29—Cl2	117.43 (18)
C10—C11—C12	118.5 (3)	C28—C29—Cl2	119.63 (17)
C10—C11—H11	120.7	C31—C30—C29	119.4 (2)
C12—C11—H11	120.7	C31—C30—H30	120.3
C13—C12—C11	121.1 (3)	C29—C30—H30	120.3
C13—C12—H12	119.5	C32—C31—C30	119.6 (2)
C11—C12—H12	119.5	C32—C31—H31	120.2
C12—C13—C8	120.4 (2)	C30—C31—H31	120.2
C12—C13—H13	119.8	C31—C32—C33	120.7 (2)
C8—C13—H13	119.8	C31—C32—H32	119.6
C15—C14—C19	116.5 (2)	C33—C32—H32	119.6
C15—C14—C3	123.4 (2)	C32—C33—C28	121.9 (2)
C19—C14—C3	120.10 (19)	C32—C33—H33	119.1
C16—C15—C14	122.2 (2)	C28—C33—H33	119.1
C16—C15—Cl1	117.7 (2)	C7A—N1—N2	107.59 (19)
C14—C15—Cl1	120.18 (18)	C8—N2—N1	115.95 (18)
C17—C16—C15	119.7 (2)	C8—N2—C3	121.56 (17)
C17—C16—H16	120.2	N1—N2—C3	110.18 (17)
C15—C16—H16	120.2	C4—N5—C20	110.11 (18)
C16—C17—C18	119.9 (2)	C4—N5—C6	111.69 (18)
C16—C17—H17	120.1	C20—N5—C6	108.06 (17)
			
N2—C3—C3A—C7A	18.3 (2)	C26—C21—C22—C23	−0.6 (4)
C14—C3—C3A—C7A	139.43 (18)	C20—C21—C22—C23	179.1 (2)
N2—C3—C3A—C4	137.96 (19)	C21—C22—C23—C24	0.5 (5)
C14—C3—C3A—C4	−100.9 (2)	C22—C23—C24—C25	0.0 (5)
C7A—C3A—C4—N5	−52.9 (2)	C23—C24—C25—C26	−0.4 (5)
C3—C3A—C4—N5	−167.63 (18)	C22—C21—C26—C25	0.2 (4)
N5—C6—C7—C27	−141.8 (2)	C20—C21—C26—C25	−179.5 (2)
N5—C6—C7—C7A	39.4 (3)	C24—C25—C26—C21	0.3 (4)
C27—C7—C7A—N1	−36.8 (3)	C7A—C7—C27—C28	172.6 (2)
C6—C7—C7A—N1	142.1 (2)	C6—C7—C27—C28	−6.1 (4)
C27—C7—C7A—C3A	147.8 (2)	C7—C27—C28—C33	−31.2 (4)
C6—C7—C7A—C3A	−33.3 (3)	C7—C27—C28—C29	153.8 (3)
C4—C3A—C7A—N1	−135.4 (2)	C33—C28—C29—C30	−0.6 (4)
C3—C3A—C7A—N1	−11.3 (2)	C27—C28—C29—C30	174.7 (2)
C4—C3A—C7A—C7	40.4 (3)	C33—C28—C29—Cl2	178.85 (18)
C3—C3A—C7A—C7	164.53 (19)	C27—C28—C29—Cl2	−5.9 (3)
C13—C8—C9—C10	−0.4 (3)	C28—C29—C30—C31	0.9 (4)
N2—C8—C9—C10	−178.2 (2)	Cl2—C29—C30—C31	−178.6 (2)
C8—C9—C10—C11	−0.6 (4)	C29—C30—C31—C32	−0.2 (4)
C9—C10—C11—C12	1.1 (4)	C30—C31—C32—C33	−0.8 (4)
C10—C11—C12—C13	−0.6 (4)	C31—C32—C33—C28	1.1 (4)
C11—C12—C13—C8	−0.3 (4)	C29—C28—C33—C32	−0.4 (4)
C9—C8—C13—C12	0.8 (3)	C27—C28—C33—C32	−175.6 (2)
N2—C8—C13—C12	178.6 (2)	C7—C7A—N1—N2	−177.40 (19)
N2—C3—C14—C15	−140.5 (2)	C3A—C7A—N1—N2	−1.7 (3)
C3A—C3—C14—C15	103.9 (3)	C13—C8—N2—N1	35.8 (3)
N2—C3—C14—C19	39.0 (3)	C9—C8—N2—N1	−146.42 (19)
C3A—C3—C14—C19	−76.6 (3)	C13—C8—N2—C3	174.33 (19)
C19—C14—C15—C16	−1.5 (4)	C9—C8—N2—C3	−7.9 (3)
C3—C14—C15—C16	178.0 (2)	C7A—N1—N2—C8	158.2 (2)
C19—C14—C15—Cl1	179.33 (19)	C7A—N1—N2—C3	15.1 (2)
C3—C14—C15—Cl1	−1.2 (3)	C14—C3—N2—C8	74.5 (2)
C14—C15—C16—C17	0.3 (4)	C3A—C3—N2—C8	−161.78 (18)
Cl1—C15—C16—C17	179.5 (2)	C14—C3—N2—N1	−144.89 (18)
C15—C16—C17—C18	1.3 (4)	C3A—C3—N2—N1	−21.2 (2)
C16—C17—C18—C19	−1.8 (4)	C3A—C4—N5—C20	−176.51 (18)
C17—C18—C19—C14	0.6 (4)	C3A—C4—N5—C6	63.4 (2)
C15—C14—C19—C18	1.1 (4)	C21—C20—N5—C4	61.7 (3)
C3—C14—C19—C18	−178.5 (2)	C21—C20—N5—C6	−176.1 (2)
N5—C20—C21—C26	59.8 (3)	C7—C6—N5—C4	−56.7 (2)
N5—C20—C21—C22	−119.9 (2)	C7—C6—N5—C20	−178.0 (2)

### Hydrogen-bond geometry (Å, °)

**Table:**

*D*—H···*A*	*D*—H	H···*A*	*D*···*A*	*D*—H···*A*
C3—H3···Cl1	0.98	2.61	3.101 (2)	111
C27—H27···Cl2	0.93	2.68	3.043 (3)	104
